# 128. Validation of a rapid diagnostics platform for detecting extended-spectrum beta-lactamase resistance in gram negative bacteremia

**DOI:** 10.1093/ofid/ofad500.201

**Published:** 2023-11-27

**Authors:** Sam Andrews, Tristan Timbrook, Mark Fisher, Brandon Tritle

**Affiliations:** UCSF Health, San Francisco, California; University of Utah College of Pharmacy, Salt Lake City, Utah; ARUP Laboratories; University of Utah Health, Salt Lake City, Utah; University of Utah Health, Salt Lake City, Utah

## Abstract

**Background:**

Extended-spectrum beta-lactamase-producing Enterobacterales (ESBL-E) bloodstream infections (BSI) are associated with increased cost, morbidity, and mortality compared with BSI by non-ESBL-producing bacteria. Phenotypic susceptibility data from blood cultures can take up to 72 hours to result, leading to delays in appropriate antimicrobial administration and unnecessary carbapenem usage. In April 2021, University of Utah (U of U) Health began utilizing the BIOFIRE® Blood Culture Identification (BCID2) Panel platform for rapid identification of organisms and resistance genes in blood cultures, including CTX-M, the most common ESBL gene in the United States. However, other genetic determinants can lead to third-generation cephalosporin resistance. Therefore, we sought to determine the positive predictive value (PPV), negative predictive value (NPV), sensitivity, and specificity of the BCID2 platform for predicting phenotypic ceftriaxone susceptibility at our institution. Additionally, we sought to determine susceptibility of ertapenem among our isolates as co-resistance has been reported amongst CTX-M containing isolates.

**Methods:**

This was a retrospective observational cohort study evaluating adult patients with bacteremia by select Enterobacterales species (Escherichia coli, Klebsiella oxytoca, Klebsiella pneumoniae group, Proteus, Salmonella). Data collection was performed utilizing the Enterprise Data Warehouse, clinical lab data, and medical chart review.

**Results:**

Between 4/1/2021 and 1/31/2023, 386 isolates (in 386 patients) of Enterobacterales were identified; the majority of isolates were *E. coli* (280, 72.5%). Ceftriaxone resistance was observed in 45 isolates (11.6%). We found that BCID2 is highly specific (100%) and sensitive (91.1%) for predicting ceftriaxone susceptibility with high NPV (98.8%) and PPV (100%) at the U of U Health. All ceftriaxone-resistant isolates were susceptible to ertapenem.

**Conclusion:**

The BCID2 platform is a highly sensitive and specific rapid diagnostic for determining ceftriaxone resistance at our institution. These findings should be confirmed at individual institutions to account for local resistance epidemiology.

**Disclosures:**

**Tristan Timbrook, PharmD**, bioMerieux: Employee|bioMerieux: Stocks/Bonds **Mark Fisher, PhD**, Copan Diagnostics: Grant/Research Support|Paratek Pharmaceuticals: Grant/Research Support

